# Morphological classification of acromial spur: correlation between Rockwood tilt view and arthroscopic finding

**DOI:** 10.1051/sicotj/2016039

**Published:** 2017-01-11

**Authors:** Pinkawas Kongmalai, Adinun Apivatgaroon, Bancha Chernchujit

**Affiliations:** 1 Department of Orthopaedics, Faculty of Medicine, Thammasat University Paholyothin Road Khlong Luang, Rangsit Pathum Thani 12121 Thailand

**Keywords:** Impingement syndrome, Acromion spur, Shape, Morphology, Arthroscopic classification, Rockwood tilt view

## Abstract

*Purpose and hypothesis*: Acromion spur is the extrinsic factor for impingement syndrome and rotator cuff tear. The Rockwood tilt view can be used to evaluate prominence of the anterior acromion, however no study has shown the correlation of findings between the Rockwood tilt view and the arthroscopic finding.

*Methods*: We developed the arthroscopic classification of acromion spur as type 1 flat spur, type 2 bump spur, type 3 heel spur, type 4 keel spur, and type 5 irregular spur. Patients with rotator cuff syndrome who underwent arthroscopic surgery were recruited. Two observers were asked to classify the type of spur from arthroscopic findings and Rockwood tilt views separately in random pattern. The prevalence of supraspinatus tendon tear was also recorded as no tear, partial-thickness tear, and full-thickness tear.

*Results*: The keel spur (33.9%) was the most common finding followed by the heel spur (27.8%). The correlation was high especially for the heel, the keel, and the irregular spur (75.47%, 74.03%, and 72.73%, respectively.) These three types of spurs have a high prevalence of full thickness of supraspinatus tendon tear.

*Conclusion*: The Rockwood tilt view can be used to evaluate the morphology of an acromion spur, especially the at-risk spur that correlates highly with the full-thickness supraspinatus tendon tear. The arthroscopic classification will also be a useful tool to improve communication between the surgeon and the guide for appropriate treatment in a rotator cuff tear patient when encountering the heel, keel, and irregular spur.

## Introduction

One of the most common causes of shoulder pain is an impingement syndrome, which may lead to a rotator cuff tendon tear. The underlying causes of such shoulder pain are still controversial between the degenerative changes in the tendons or the compression by the acromion spur. In 1972, Neer [1] emphasized the role of the anterior acromion in rotator cuff disorders and described anterior acromioplasty as a surgical treatment for chronic impingement syndrome [2]. After that, Bigliani et al. [3] reported a clinical correlation between the acromial type and the rotator cuff tear. More recently, Balke et al. [4] reported the lack of morphological characteristics of degenerative rotator cuff tears in traumatic cases. Their findings support the theory of external mechanical compression as a major cause of degenerative rotator cuff disease.

The traditional method of classifying the acromion is based on the shape of its undersurface in sagittal plane. First described by Bigliani et al. [5] in 1986, the acromion can be classified as type I (flat), type II (curved), or type III (hooked). There were many articles that demonstrated a close correlation of the hook acromion with rotator cuff disease [6, 7]. However, the difficulty in obtaining true supraspinatus outlet view radiographs and the poor inter-observer reliability have brought a significant question for this classification [8–10].

In 1984, Kitchel et al. [11] reported on the use of a special X-ray film of the 30° caudal tilt of the shoulder in 200 patients with shoulder impingement syndrome to evaluate the prominence of the anterior acromion. They demonstrated that this special view was easy to take and that the findings were reproducible. In 1991, Ono et al. [12] also confirmed that the 30° caudal tilt view showed the exact shape of the spur encountered at operation. However, they did not show the correlation of this finding clearly.

An understanding of the morphology of the acromial spurs before the operation could be beneficial in terms of the diagnosis and management of impingement syndrome and rotator cuff tears. This article describes our research into the question “Does the shape of the bony spur that seen from the 30° caudal tilt view be the same as that encountered at operation?” We hypothesized that the 30° caudal tilt view or the Rockwood tilt view can be used to demonstrate the shape of the acromion spur encountered at operation. Furthermore, the prevalence of the rotator cuff tear that accompanies the variable shape of the acromion spur was also studied.

## Material and methods

A retrospective review was conducted after approval was obtained from the local institutional review board. Patients with rotator cuff syndrome who underwent arthroscopic surgery from July 2012 to June 2014 at the Thammasat University Hospital were recruited. All of these patients had undergone refractory to fully conservative treatment for at least six months.

Exclusion criteria were patients with previous shoulder trauma, previous surgery on either shoulder, inflammatory arthropathy, glenohumeral osteoarthritis, adhesive capsulitis or insufficient quality of the intra-operative arthroscopic video.

**Figure 1.**
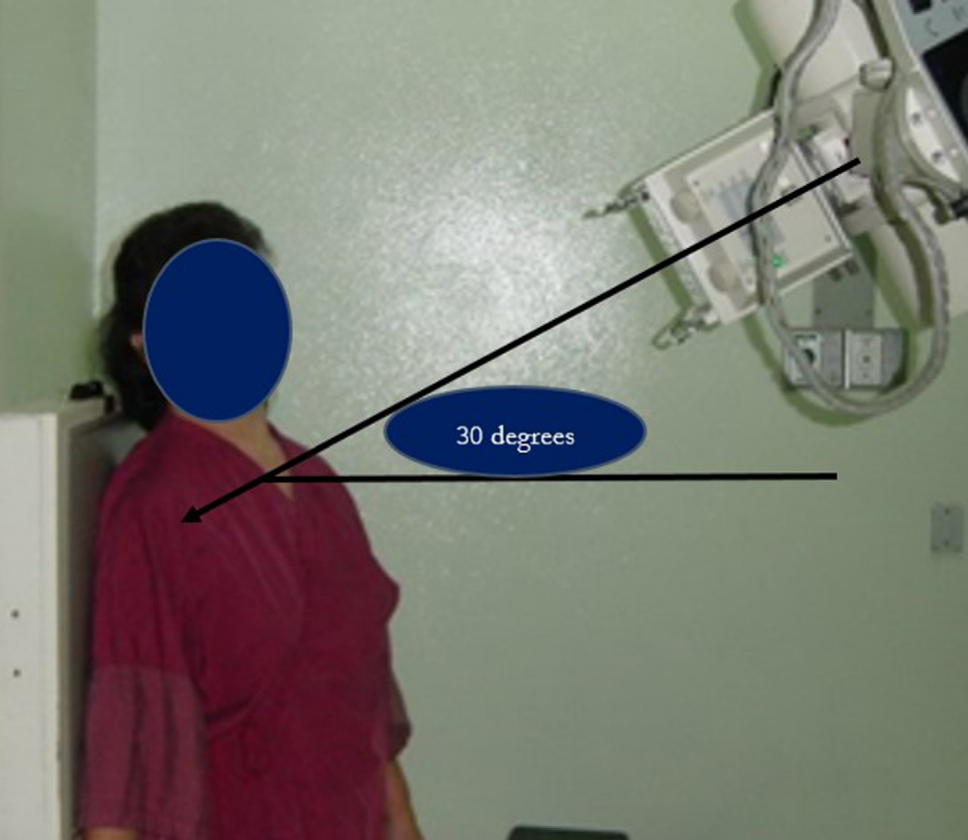
Standard position for the Rockwood tilt view film. The film was placed behind the shoulder of the patient, who was standing 35 cm between the cassette and the source. The X-ray beam was projected from the anterior direction at a 30° tilt from superior to inferior.

The radiographs were performed by three radiology technicians who were trained to standardize the quality of the radiographs. The distance between the cassette and the X-ray beam was 35 cm for all X-rays. Radiographs of the 30° caudal tilt view of the affected shoulder were obtained. The film was placed behind the shoulder of the patient, who was standing and the X-ray film was projected from the anterior direction at a 30° tilt from superior to inferior (Figure 1).

Due to the historically poor inter-observer reliability of acromial spur classifications, we developed a new classification of acromial spur for our study. We believe that the morphology of the acromial spur from the arthroscopic finding was the true morphology, so we have developed this classification primarily based on the arthroscopic finding. Based on an arthroscopic video in the beach chair position intra-operatively, we captured the picture of the acromial spur from the camera in posterior portal. For the right shoulder, the camera post was pointed to 2 o’clock. For the left shoulder the camera post was pointed to 11 o’clock.

Two separate training sessions were conducted to allow for the clarification and interpretation of the classification systems used in this analysis as described in Table 1.

**Table 1.** Describes the arthroscopic morphological classification of the acromion spur with the arthroscopic picture for each type.

Acromion spur type	Description	Picture
1. Flat	Spur less than 2 mm from the inferior edge of the distal clavicle.	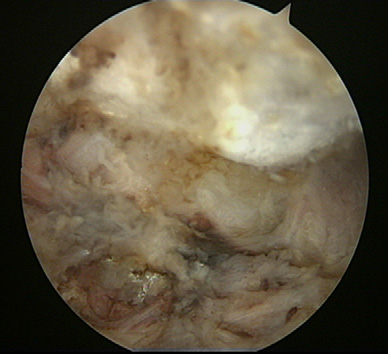
2. Bump	Bump shape spur more than 2 mm from the inferior edge of the distal clavicle.	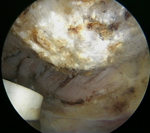
3. Heel	Quadrangular shape spur more than 2 mm from the level of the distal clavicle (like the heel of the shoe).	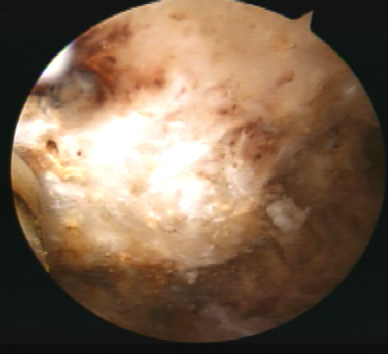
4. Keel	Sharp spur more than 2 mm from the level of the distal clavicle with central-downward facing like a sailboat’s keel.	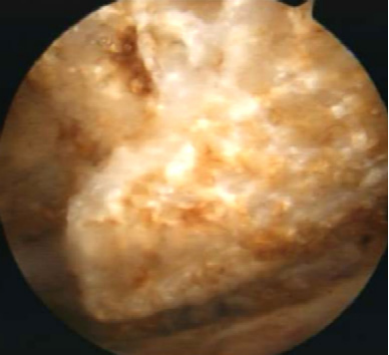
5. Irregular	Spur of the acromial side protruding more than 2 mm from the level of the distal clavicle with an irregular shape.	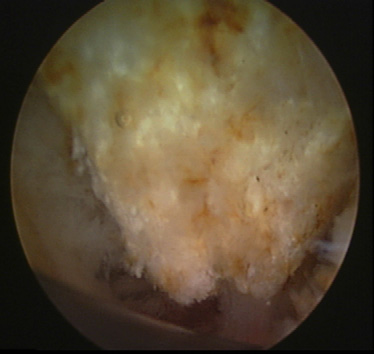

The review process took place after the training sessions and there was no further collaboration after the analysis had begun. In order to validate the classification system, we analyzed the intra- and inter-observer reliability of the system. Two independent observers, who were fellowship-trained sports surgeons, blinded to the clinical and magnetic resonance imaging (MRI) data were asked to classify the selected arthroscopic pictures in a random order and at three-week intervals in order to establish the intra-observer reliability. The inter-observer reliability was also analyzed from the results gathered in the first time of each observer. The condition of supraspinatus tendon was also recorded from the operative note as no tear, partial thickness tear, and full-thickness tear.

One month later, they were asked to review the type of acromial spur from the 30° caudal tilt view for each patient in random pattern. If the results from arthroscopic pictures and the 30° caudal tilt view by the two observers are different, the discussion for consensus will be done and used to compare to the arthroscopic classification.

## Statistics

We estimated the sample size for one-sample comparison of proportion to the hypothesized value. We used *p* = 1.000, where *p* is the proportion in the population. The assumptions are as defined below: *α* = 0.05 (two-sided), power = 0.80, and alternative *p* = 0.90. The estimated required sample size is seven patients per type of classification. Because of the new classification that we developed, we do not know the normal distribution of each type so we tried to collect the cases as much as we could in the time period.

Regarding the calculation of reliability, the weighted Cohen kappa (*k*) values were calculated. The *k* values were interpreted as described by Landis and Koch [13]. The *k* values of .81 to 1.0 demonstrated excellent agreement, .61 to .80 substantial agreement, .41 to .60 moderate agreement, .21 to .40 fair agreement, and 0 to .20 slight agreement.

## Result

From July 2012 to June 2014, the medical records documented 252 patients with a rotator cuff syndrome who failed conservative treatment and underwent arthroscopic surgery at the Thammasat University Hospital. Seventy-two patients were excluded for the following reasons: previous shoulder trauma (8), previous surgery on either shoulder (9), inflammatory arthropathy (3), glenohumeral osteoarthritis (6), adhesive capsulitis (20), or insufficient quality of the intra-operative arthroscopic video (26). This results in a total of 180 patients for the analysis. In this study, 44.8% of patients were male. The left shoulder was involved in 48.4% of the cases. The mean age of the patients was 57 years (range, 35–79).

A statistically significant intra- and inter-observer reliability was noted for the arthroscopic classification of the acromial spur. It was found to have an excellent agreement as shown in Table 2.

**Table 2.** Describes the reliability of arthroscopic morphological classification of the acromial spur.

	Reliability (kappa value)		
	Intra-observer		Inter-observer
	Observer 1	Observer 2	
1. Flat	0.98	0.98	0.98
2. Bump	0.97	0.97	0.93
3. Heel	0.93	0.92	0.89
4. Keel	0.92	0.91	0.91
5. Irregular	0.94	0.97	0.92

**Figure 2.**
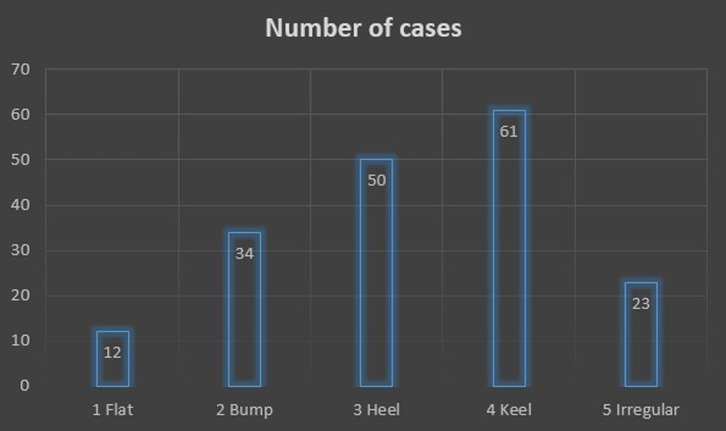
Distribution of the acromion spur type classified from the arthroscopic picture.

For the distribution along morphological classification, the result is shown in Figure 2. We have found the flat type in 12 patients (6.6%). The bump type was found in 34 patients (18.9%). The heel type was found in 50 patients (27.8%) and the most common type we have found is the keel type with 61 patients (33.9%). The irregular type was found in 23 patients (12.8%).

Keeping arthroscopic finding as the standard reference, the result of positive predictive value (PPV) is shown in Table 3. The flat and bump spur has low PPV which is 29.41% and 54.55%, respectively. The PPV for the heel, keel, and irregular spur was high which is 75.47%, 74.03%, and 72.73%, respectively.

**Table 3.** Describes the correlation of the type of the acromion spur between the arthroscopic finding and the Rockwood tilt view.

Film	Type 1	Type 2	Type 3	Type 4	Type 5
Scope	Flat	Bump	Heel	Keel	Irregular
Type 1	5	12	0	0	0
Flat	29.41	70.59	0.00	0.00	0.00
Type 2	6	12	3	0	1
Bump	27.27	54.55	13.64	0.00	4.55
Type 3	1	7	40	3	2
Heel	1.89	13.21	75.47	5.66	3.77
Type 4	0	1	7	57	12
Keel	0.00	1.30	9.09	74.03	15.58
Type 5	0	2	0	1	8
Irregular	0.00	18.18	0.00	9.09	72.73

**Figure 3.**
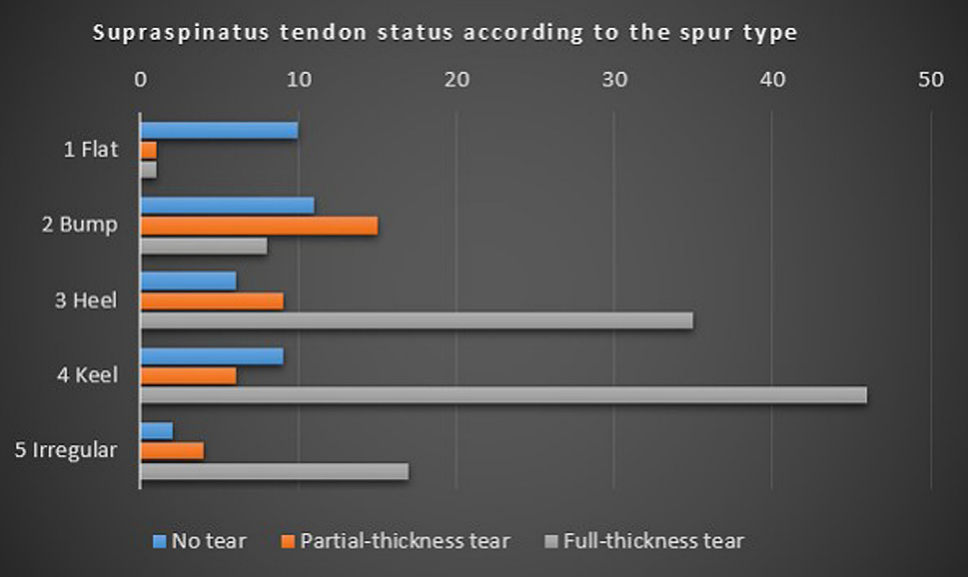
The prevalence of the supraspinatus tendon tear along the morphological classification.

The prevalence of the supraspinatus tendon tear along the morphological classification is shown in Table 4 and Figure 3. The flat spur and the bump spur have only 8.33% and 23.53% of full-thickness supraspinatus tendon tear, respectively. In contrast, the heel spur, the keel spur, and the irregular spur have 70%, 75.41%, and 73.91% of full-thickness supraspinatus tendon tear.

**Table 4.** Describes the prevalence of the supraspinatus tendon status according to the arthroscopic morphological classification.

Spur type	Supraspinatus tendon status		
	No tear	Partial-thickness tear	Full-thickness tear
1. Flat	10	1	1
	83.33	8.33	8.33
2. Bump	11	15	8
	32.35	44.12	23.53
3. Heel	6	9	35
	12.00	18.00	70.00
4. Keel	9	6	46
	14.75	9.84	75.41
5. Irregular	2	4	17
	8.70	17.39	73.91

## Discussion

Since 1972, Neer found that rotator cuff tears are caused by the impingement of proliferative acromial spurs upon the rotator cuff tendons. After that, several authors have investigated the structure of the acromion in patients with a rotator cuff disease [1, 14–16].

However, the association between acromial morphology and rotator cuff tears remains to be fully established. The classical method of classifying the acromion shape was described by Bigliani et al. [5]. Balke et al. [17] supported the finding that the hook acromion is the predisposing factor to degeneration of supraspinatus tendon by publishing that, compared to the control group, significant portion of patients with subacromial pathology had the hook acromion. However, the result of Jacobson et al. [9] and that of Zuckerman et al. [18] reported the poor intra- and inter-observer agreement of this classification. Hamid et al. [19] also confirmed that Bigliani acromial morphology classification system lacked inter-observer reliability despite a standardized fashion with a precise radiographic protocol by specially trained staff.

Several studies suggested several effects of the acromial spur on the shoulder pain. It is not only the reduction of the subacromial space, but also the shape of the acromion that increases the risk of rotator cuff tears [20–22]. For this purpose, the acromial morphology must be evaluated carefully before the operation to ensure that the real pathology of the patients is being addressed and to find patients who may benefit from surgical intervention. In addition, it is also important to determine the exact shape of any bony spur of the anterior acromion, not only because such a spur is the external cause of the impingement syndrome but also because it is the direct target of the acromioplasty procedure.

**Figure 4.**
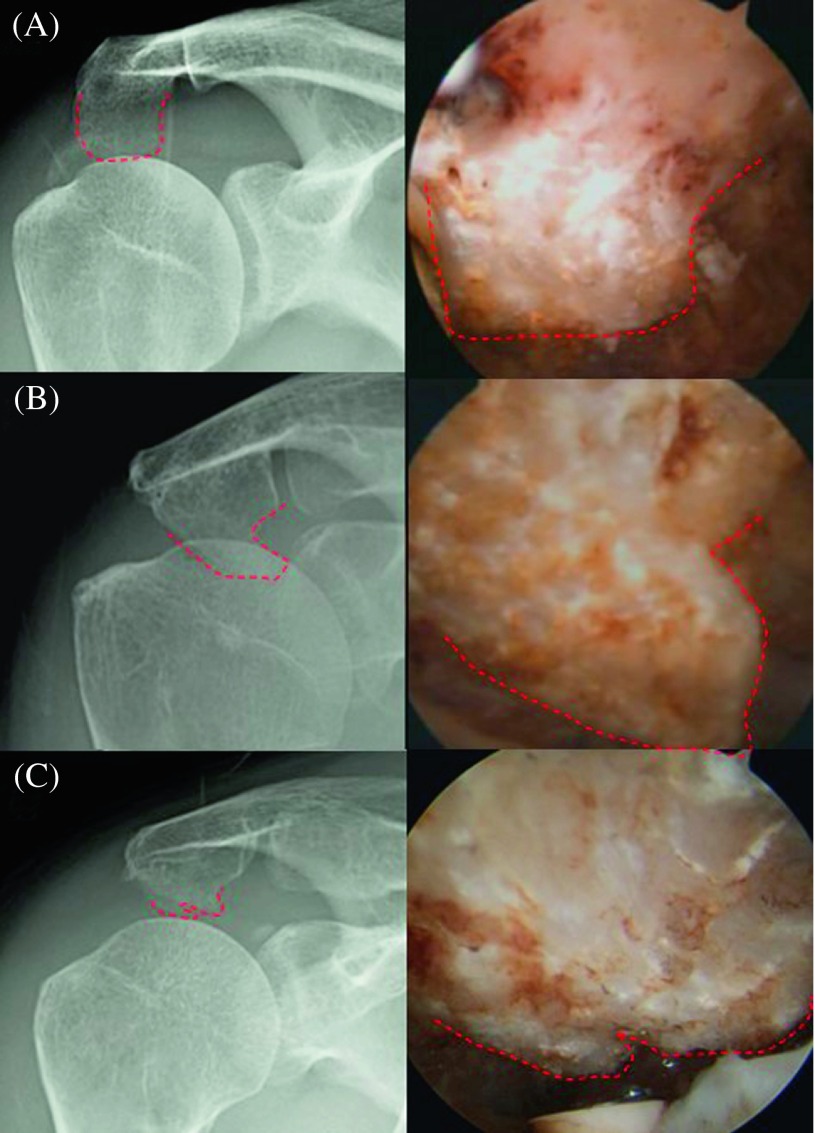
The example of the “at-risk spur” from arthroscopic classification compared to the Rockwood film. (The heel spur is in inset (A), the keel spur is in inset (B), and the irregular spur is in inset (C)).

Using 180 cases, the most common type of acromial spur classified by arthroscopy is the keel spur, which we found in 33.9% of our patients. First described by Tucker and Snyder [23], the keel refers to a central, longitudinal, downward sloping spur on the acromial undersurface. Patients with a keel spur are at significant risk of bursal-sided torn as well as full-thickness rotator cuff tears. Our results are in agreement with their results. This is followed by the heel spur, which is a quadrangular-shaped spur like the heel of a shoe. This represents 27.8% in our series of 180 cases. This result is similar to the report of Oh et al. [24], whose data suggest that the most common heel-type spur might be a risk factor for full-thickness rotator cuff tears. We also first described the irregular shape spur, which is the spur that protrudes from the acromial side by more than 2 mm below the inferior edge of the distal clavicle with an irregular surface. Although this spur type may not be commonly found, they do account for the high prevalence of rotator cuff tear. Therefore, we suggest to call the type 3–5 as the “at-risk” acromion spur, or the spurs which account for the high prevalence of the full-thickness supraspinatus tendon tear (Figure 4). The flat and bump acromion spur were accounted for only 6.6% and 18.9%, respectively. These types were also associated less with full-thickness supraspinatus tendon tear.

Kitchel et al. [11] and Ono et al. [12] reported on the use of the Rockwood tilt view to evaluate prominence of the anterior acromion. The 30° in the caudal direction aligns the X-ray beam parallel to the inferior surface of the acromion, allowing the inferiorly projecting spurs to be more easily visualized. Cone et al. [25] also support this theory by stating that the delineation of subacromial spurs can be improved by tilting the beam 30° caudally.

We found the high correlation between arthroscopic finding and Rockwood tilt view for heel, keel, and irregular spur. Because these three types of spurs account for the high prevalence of full-thickness supraspinatus tendon tear, we can imply the importance of Rockwood tilt view to determine the at-risk type of acromion spur before the operation. The correlation for the flat and bump spur is low, which may be the weakness of the Rockwood tilt view. The explanation is that the size or the thickness of acromial spur on the film, which is certainly subject to projection errors arising not only from the position of the patient but also from the direction of the radiographic beam, may mislead the true size of the acromion spur. However, these types of spurs were accounted for in the minority population of rotator cuff tear patients and may not have any clinical significance. In contrast to type 3–5, which we classified mainly by the shape, these at-risk spur types could be evaluated pre-operatively by the Rockwood tilt view.

Miller et al. [26] suggest that the radiographs to evaluate the patients with impingement syndrome should include an anteroposterior view, axillary lateral view, and supraspinatus outlet view. However, many of the abovementioned authors suggested that the classification of acromial spur by supraspinatus outlet view may not be enough. From our results, we would like to suggest adding Rockwood tilt view to this series, as there may be more benefits to classify the at-risk acromion spur from the Rockwood tilt view.

**Figure 5.**
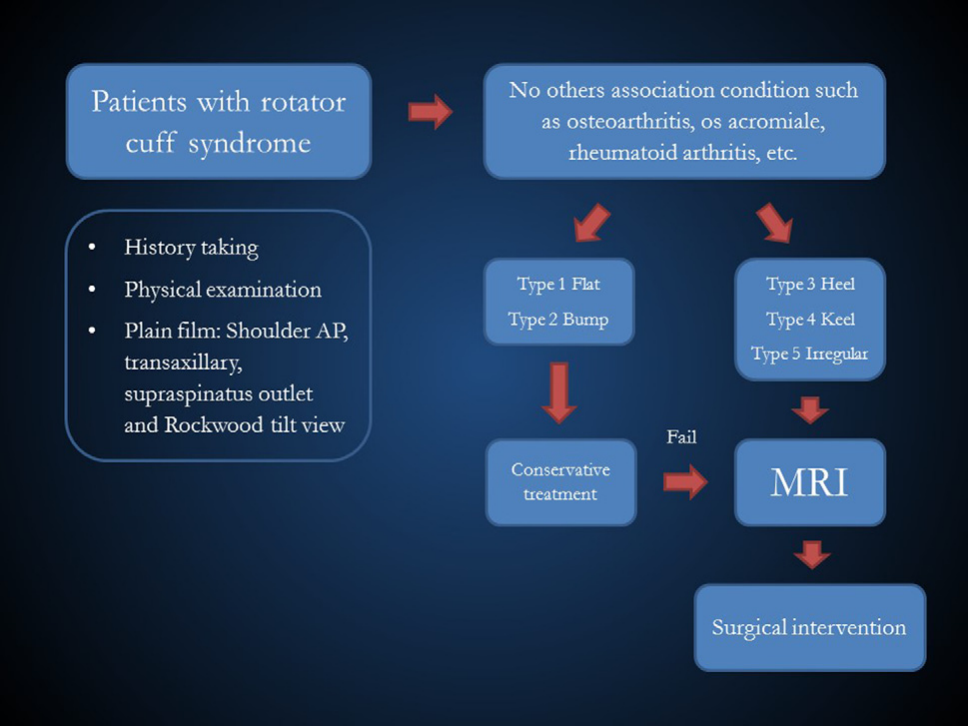
The diagram for the evaluation of patients with rotator cuff syndrome which utilizes the arthroscopic classification as the factor to consider.

The arthroscopic classification of acromial spur morphology has several implications, which are not only to improve the communication between the surgeons themselves, but also to guide in the appropriate treatment in a rotator cuff tear patient when encountering the heel, keel, and irregular spur. In the field of clinical evaluation, if the patients present to the clinic with an impingement syndrome with these “at-risks” spurs seen from the Rockwood tilt view, we can assume that these patients may have supraspinatus tendon tear. Therefore, these patients may need more aggressive evaluation such as early MRI in the first or the second visit to find the reality of the tear, the presence of the retraction, or the fatty degeneration. (Figure 5) In the field of surgery, as we already mentioned that while the role of acromioplasty is still a controversy, we suggest adding this procedure if we encounter the at-risk spur. This is not only to treat the cause of impingement syndrome, but also to increase the space available to do the entire work in arthroscopic surgery in the subacromial space too.

## Limitation

This study had some limitations due to a study design that was the retrospective analysis. We did not explore the temporal changes of subjects through the time period. Therefore, we may not summarize that the association of acromion spur with rotator cuff disease is a direct causal relationship. Furthermore, we have not determined the prevalence of the at-risk acromion spur in an asymptomatic patient population. In addition, both observers in this study are from the same center, therefore, there may be some bias in interpreting the reliability of our arthroscopic classification. We tried to reduce this error by setting the separate session of each author to interpret, and we planned to recruit more observers from different centers and patients in the future. The last one is that no postoperative evaluation has been provided, and the current literature does not support the use of acromioplasty in the surgical treatment of rotator cuff disease. None of the studies recommend against this procedure. Our study will guide the surgeons in the morphology of the acromion spur when they need to perform acromioplasty, so as to increase the space for the rotator cuff repair.

## Conclusion

The Rockwood tilt view can be used to evaluate the morphology of acromion spur especially the at-risk spur that correlates highly with the full-thickness supraspinatus tendon tear. The “at-risks” acromial spur consists of the heel, the keel, and the irregular spur. The keel spur is the most common type in our series which is followed by the heel spur. The arthroscopic classification will also be a useful tool to improve communication between the surgeon and the guide in the appropriate treatment in a rotator cuff tear patient when encountering the “at-risks spur”.

## Conflict of interest

The authors declare no conflict of interest in relation with this paper.

These authors and their immediate family did not receive any financial payments or other benefits from any commercial entity related to the subject of this article.
